# Spectral interlocutions and the politics of unfinished: Buddhism, haunting, and memory in Karunatilaka’s *The Seven Moons of Maali Almeida*

**DOI:** 10.3389/fsoc.2025.1712299

**Published:** 2026-01-06

**Authors:** Neethu Mary Humphry, Ajit I

**Affiliations:** School of Social Sciences & Languages, Vellore Institute of Technology, Chennai, Tamil Nadu, India

**Keywords:** Buddhism, hauntology, restorative justice, memory, spectrality, Sri Lanka

## Abstract

Shehan Karunatilaka’s *The Seven Moons of Maali Almeida* (2022) offers a reimagined depiction of the Sri Lankan civil war aftermath in the context of restorative justice. It offers a discussion of how haunting serves as a sociological process for truth-telling, recognition, and reconciliation within post-war Sri Lanka. Using a theoretical approach rooted in Derrida’s hauntology, Avery Gordon’s notion of haunting, and Buddhist cosmology in the sociology of literature framework, this research examines how the novel reworks the afterlife as a site of moral and collective accountability. The war photographer, Maali Almeida, who died during the civil war, is a witness. Through his ghostly presence, he forces the living to reveal hidden atrocities and acknowledges collective damage. His haunting challenges political amnesia. And it represents how memory, justice, and healing are indistinguishably related in a violence-plagued society. Finally, by imagining haunting as a metaphor and a means of restorative justice, the paper argues that Karunatilaka’s novel does not imagine reconciliation through retribution or denial, but in terms of truth-telling, memory, and ethical liberation.

## 1 Introduction

In his seminal work, *Spectres of Marx*, Jacques Derrida conceptualises spectrality as the presence of what ought to be absent. It is what history has endeavoured to forget, resisting fixed categorisation and stability. This paradoxical presence destabilises linear temporality and fixed identity. This ambiguity permits the past to infiltrate the present. Spectrality manifests in various forms, such as historical spectres that reveal the ghosts of suppressed or erased histories. Psychic phantoms internalise traumas or unconscious drives, and temporal spectres that disrupt linear time by crawling upon the present or future. Whereas, ontological spectres blur the boundaries between life and death, presence and absence. And political ghosts are figures or events that haunt collective memory. Central to this paper is the concept of political spectrality, and here haunting functions as a subversive force that exposes historical silences, censored traumas, and ideological erasures. The ghost thus becomes a conduit for unspoken suffering. This reveals the ethical shortcomings of dominant historical narratives. Trauma is an experience that returns in fragments and symptoms, and the ghost in post-conflict literature reflects this experience. It is not merely a character but a performative figure through which trauma articulates itself. As Davis (2005) says, “Attending to the ghost is an ethical injunction insofar as it occupies the place of the Levinasian Other: a wholly irrecuperable intrusion in our world, which is not comprehensible within our available intellectual frameworks, but whose otherness we are responsible for preserving.” Consequently, spectrality offers an alternative archive of memory by resisting linear chronology. Thus, it embraces multiplicity, contradiction, and uncertainty.

Restorative justice emphasises the collective dimension of trauma. It recognises that unacknowledged violence and silenced memories destabilise not only individuals but also entire communities. In *The Seven Moons of Maali Almeida*, haunting performs a similar role. The ghost of Maali, the restless spirits of riot and war victims, insist upon acknowledgement of harm. They refused the enforced amnesia imposed by the state. In numerous novels, particularly in Shehan Karunatilaka’s *The Seven Moons of Maali Almeida* (2022), spectrality is a crucial narrative device to depict atrocities and traumatic events. This Booker Prize-winning novel is groundbreaking in that it utilises elements of political satire and the ghost story genre to scrutinise the contentious history of post-independence Sri Lanka. The year 1983 is indelibly etched in Sri Lankan history due to an anti-Tamil pogrom, a state endorsement, which led to widespread bloodshed, and has since come to be known as ‘Black July.’ “The ethnic strife between the Sinhala government and the Tamils was ignited by the Standardisation Act of 1972, the destruction of the Jaffna Public Library in 1981, and the 1983 anti-Tamil riots” (Jenisha and Boopathi, 2024, p. 83). It cost a thousand deaths, and many Tamils were displaced. A photograph taken by the photojournalist Chandragupta Amarasinghe depicts “a naked Tamil man cowering on a bench while several laughing young Sinhalese men swing their feet in his direction” (Ganeshananthan, 2023, para. 2), and it shows the escalation of the situation. In the novel, Maali Almeida, a war photographer, captures a similar image. To avoid issues, he hides it within his cache of politically sensitive photographs. The narrative follows the ghost of Maali as he unravels the mystery of his death, which happened during the civil war in the late 1980s. Throughout his journey, he encounters a cache of photographs that document state and paramilitary brutality, as well as hidden truths. The presence of a spectral protagonist offers a deep reflection on memory by showing political violence and trauma in their long-term effects.

In recent years, Sri Lankan Anglophone fiction has increasingly focusedon themes of one’s national memory and about the unspoken aspects of history, especially regarding the aftermath of the civil war. Works such as Shyam Selvadurai’s *Funny Boy* (1994) and *Cinnamon Gardens* (1998), Nayomi Munaweera’s *Island of a Thousand Mirrors* (2012), and Anuk Arudpragasam’s *The Story of a Brief Marriage* (2016) exemplify this focus by addressing the enduring psychological and political ramifications of the conflict. However, Karunatilaka’s novel distinguishes itself through the innovative use of Maali’s ghost for narration as well as witnessing. As Berger (1972) articulates, “the art of the past is being mystified because a privileged minority is striving to invent a history which can retrospectively justify the role of the ruling classes” (p. 7). Similarly, history in this context is manipulated to fit popular narratives. The narrative is in the second-person point of view, which is a potent narrative strategy. From this perspective, the silenced, the missing, and the forgotten re-emerge to challenge the nation’s dominant narratives that distort history. As Gordon (1997) said, “Haunting is not the same as being exploited, traumatised, or oppressed, although it usually involves these experiences or is produced by them” (p. 19). Consequently, Maali transcends the conventional literary depiction of a ghost, embodying a persistent social memory that resists being forgotten. Therefore, the paper contributes to reassessing silenced histories through spectral interlocutions and to analysing restorative justice by demanding the communal acknowledgment of harm.

## 2 Materials and methods

The study employed a qualitative sociological framework, combining narrative and discourse analysis with restorative justice perspectives, to examine Shehan Karunatilaka’s *The Seven Moons of Maali Almeida.* Framing in the sociology of literature, this study portrays this novel as a cultural document. It encodes and reflects the social structures, collective anxieties, and moral dilemmas of post-conflict Sri Lanka. As Goldmann (1975) says, literary works are “social facts that express collective meanings.” Whereas Bourdieu (1993) argues that literature constitutes a “field of cultural production” shaped by power, ideology, and history. The aim is to use literary analysis as a sociological method. Thus, treats fiction as a tool for social inquiry that reveals memory, cultural imaginaries, and societal responses to war. Through spectral interlocution, the novel’s haunting and Buddhist cosmologies are analysed to show post-war moral and social landscapes of social reality.

### 2.1 Theoretical framework

This paper applies three intersecting frameworks, such as spectral theory, restorative justice, and the sociology of literature, to closely analyse *The Seven Moons of Maali Almeida.* Spectrality engages with Derrida’s notion of “hauntology,” as introduced in *Spectres of Marx* (Derrida, 1993). This theoretical framework allows for the interpretation of ghosts not merely as remnants of the past but also as potent indicators of unresolved issues carried forward from the past to the present. Avery Gordon’s *Ghostly Matters* (1997) is a concept from the sociological domain, and it posits that ghosts manifest when “the trouble they symptomize is no longer being contained or repressed.” By combining Derrida’s hauntology with Avery Gordon’s sociological concepts of haunting, this paper explores the question of how spectrality functions as a tool to interrogate history and memory in Buddhist Sri Lanka. Finally, it questions how the ghosts in this novel function as interlocutors of restorative justice in Buddhist Sri Lanka. In this context, Maali’s spectral journey serves as a sociological allegory, wherein literary fiction is employed as a tool for social inquiry, unveiling suppressed histories, silenced voices, and the processes through which moral and political reckonings are negotiated. The novel reimagines the ghost story as a critical form of political remembrance, utilising haunting as both a narrative mode and a sociological lens.

## 3 Discussion

Restorative justice is a justice philosophy that focuses on repairing the harm caused by the crime rather than punishment. It never treats crime as an offence against the law, whereas crime is considered a breach of conduct in relationships among the victims, offenders and communities. Thus, restorative justice aims to help the affected to acknowledge harm and create a space to realise what meaningful repair looks like, and this is exactly what Braithwaite (2002a) said, “because crime hurts, justice should heal. And punishments that obstruct healing by insisting on adding more hurt to the world are not justice.” Traditions across different cultures show that the purpose of justice is not to inflict pain but to give a chance for the offender to repent, and for instance, the Christian parable of the Prodigal Son conveys the idea that wrongdoing can give the possibility of reconciliation rather than a retributive response. “The ancient Palestinian restorative justice institution of the Sulha, still practised in Galilee today, is one of the richest survivals of the ideal of using the lesser evil of crime to build the greater good of a loving community” (Braithwaite, 2002b). The priority list of values of restorative justice includes non-domination, empowerment, respectful listening, etc., where non-domination is the base of all. And thus, restorative justice values “healing rather than hurting, moral learning, community participation and caring, respectful dialogue, forgiveness, responsibility, apology, and making amends” (Braithwaite, 2002b). These principles make restorative justice a vessel for healing communities fractured by trauma, including the wounds of post-war life.

Crime stands as an offence against the state or a breach of law as per the retributive justice paradigm, where the one who violates it should be punished and considered an outcast or unwanted in society. Recently, retributive justice has been replaced by restorative justice as criminology has developed and started to rehabilitate offenders and reintegrate them into society as law-abiding citizens. “Restorative justice is a way of repairing the social fabric that is damaged through violence created by a protracted social conflict. It brings about the personal healing of survivors, the reparation of past injustices, the building or rebuilding of nonviolent relationships between individuals and communities” (Jayathilaka, 2020); thus, it focuses on the reintegration of both the victim and the offender into society. The framework aligns with the spectral politics depicted in Karunatilaka’s *The Seven Moons of Maali Almeida*. The “In Between” serves as a liminal space akin to a restorative justice circle, where ghosts, representations of silenced and marginalised histories, reappear to seek acknowledgement. Restorative justice works here in a way that the spirits of the people who are dead are getting a chance to know how and why they are dead. These spectres highlight the epistemic injustices of Sri Lanka’s violent history. Suppression of testimony, the erasure of memory, and the systematic refusal to mourn are explicitly portrayed through spectral voices. By forcing the living to confront these voids, the ghosts enact “haunting,” where Gordon describes it as the unresolved matters of the deceased that disturb the present and demand ethical accountability. Along with restorative justice, haunting functions as both a metaphor and a method. It represents a spectral form of counter-memory that challenges the retributive logic of silence and punishment, instead for dialogue, testimony, and the ethical labour of remembrance.

The sociological dimension of *The Seven Moons of Maali Almeida* resonates with international attempts at post-conflict reconciliation. The Troubles (1968–1998) in Northern Ireland, such as Sri Lanka’s civil war, were characterised by ethnic–religious estrangement, asymmetrical violence, and an extended shadow of mutual mistrust between former foes. Since the Good Friday Agreement of 1998, healing initiatives like the Healing Through Remembering project and Theatre of Witness (2013) employed testimonial and shared stories to enable victims and perpetrators to coexist in modest, interdependent communities. In a parallel with Northern Ireland, where testimony and storytelling became necessary practices of cohabitation between sectarian groups, Karunatilaka’s novel shifts haunting into a story of common acknowledgement. As Rwanda’s Gacaca courts, which placed public recognition first as the foundation for healing, the ghosts in the novel force confession and remembrance. As Hankel (2019) writes, the Gacaca system aimed to “(a) identifying the truth about what happened during the genocide, (b) speeding up the genocide trials, (c) fighting against the culture of impunity, (d) contributing to the national unity and reconciliation process, and (e) demonstrating the capacity of the Rwandan people to resolve their own problems,” encapsulated in the maxim, “Truth heals—If we say what we have seen, if we confess what we have done, then our wounds will be healed.” And, as in Argentina, where the Madres de Plaza de Mayo opposed state-imposed silence through the public mourning and commemoration of the “disappeared,” Karunatilaka’s ghostly presences transform memory into resistance, insisting that justice begins with acknowledgment.

### 3.1 Sri Lanka and Buddhist cosmologies

Theravāda Buddhism was formally introduced to Sri Lanka in the 3rd century BCE by Mahinda Thera, son of Emperor Ashoka, during the reign of King Devānāmpriya Tissa. It emerged as the predominant religious tradition. Thus, it influenced Sri Lanka’s cultural, political, and spiritual identity for more than two millennia. Theravāda, meaning “School of the Elders,” prioritises the preservation of the Pāli Canon as the authoritative scripture. It emphasises individual liberation (Nirvāṇa) through ethical conduct, meditation, and wisdom, while rejecting Mahāyāna sutras and bodhisattva cosmologies. As Uyangoda (2018) noted, “The Sri Lankan Buddhist intellectual culture did not possess a conceptual apparatus, a vocabulary, for democracy and social equality to articulate the meaning of that ‘rebellion’ in socially egalitarian terms.” Sri Lankan Buddhism underwent six phases of modernisation, including institutional rebellion. And in caste-based reform movements within the Sangha, they challenged upper-caste dominance and cultural assertion. But Buddhist-Christian debates fostered a collective Sinhala Buddhist identity. The metaphysical turn, characterised by engagement with Theosophy and Orientalist scholars, redefined Buddhism as a world religion. Within the context of Sri Lankan Buddhism, the concept of the ghost or preta is not foreign but is deeply embedded in religious and cultural narratives. In Theravāda Buddhist cosmology, death is a liminal transition within samsara. The ghosts, those who die violently or without proper rites, embody unresolved karma requiring ritual acknowledgement by the living to facilitate their transition. Similarly, restorative justice asserts that communities must recognise unresolved harm to restore equilibrium, as wherein Theravāda Buddhism, neglecting ritual and ethical duties towards the deceased perpetuates imbalance. In Theravāda Buddhism, ghosts symbolise unresolved karma, whereas in restorative justice discourse, unresolved harm manifests as a haunting that necessitates community reconciliation. This sheds light on how Buddhist cosmology can act as an implicit moral code, a metaphysical system of justice that attempts to restore equilibrium through recognition of transgression. In *The Seven Moons of Maali Almeida*, haunting is a juridical metaphor: the restless dead insist on redress in much the same way as claimants in a restorative justice procedure. Karunatilaka thereby translates Buddhist conceptualisations of karmic reparation into the language of sociology, in which the ghostly is a way of speaking about moral responsibility and public reconciliation transmurally of formal law. This novel reconceptualises haunting as a political demand for community-level accountability. In this context, ghosts are not merely remnants of karma but also representations of collective trauma. This emphasises truth-telling and acknowledgement as basic prerequisites for any kind of healing.

Shehan Karunatilaka is one of the noted writers among the contemporary authors of Sri Lanka. He is acclaimed for his blend of satirical wit and incisive political commentary. He was raised in Colombo and educated in both Sri Lanka and New Zealand, and his experiences in advertising, screenwriting, and journalism have contributed to his sharp observational style and experimental approach. His work often reveals the horrors of Sri Lankan political discourse, mixing with humour, irony, and magical realist techniques to portray reality. He gained international recognition with his debut novel *Chinaman: The Legend of Pradeep Mathew*, a metaphysical narrative about a reclusive sports journalist’s quest to uncover the mysterious disappearance of a Sri Lankan cricketer. “Chinaman” addresses postcolonial identity, corruption, and the politics of truth-telling by subtly referring to the island’s ethnic tensions and civil unrest. His second novel, *The Seven Moons of Maali Almeida*, draws on overlapping conflicts, including the state-LTTE war and the JVP insurrection. It is through the ghost of Maali, that the unveiling of state and military crimes takes place. Thus, it transforms the ghost story into a form of political testimony, a chorus of voices silenced in life. The line “She tells you that she wandered for a thousand moons before she found peace. That many of the victims of the 1983 riots are still roaming the In Between” (Karunatilaka, 2022) accentuates how the victims of Sri Lanka’s ethnic violence continue to exist. The restless presence is felt because their deaths have not been fully acknowledged or mourned. The “wandering” of the riot victims symbolises the persistence of collective trauma and suppressed memory. This aligns with Gordon’s perception that ghosts return when what they evince is repressed or denied.

*The Seven Moons of Maali Almeida*, set against the backdrop of political unrest and violence in Colombo in late 1989 narrates the story of a war photographer. Maali after death, awakens in the afterlife, i.e., “the In Between,” with no recollection of his death. Given 7 days or seven moons to investigate his demise, Maali aims to guide his friends to a secret cache of photographs documenting atrocities committed by various groups, evidence that could identify the powerful individuals responsible for his assassination. Derrida posits that “a spectre is always a revenant. One cannot control its comings and goings because it begins by coming back” (p. 11). The afterlife exists as a disorganised bureaucratic world inhabited by insurgent ghosts, starving ghosts, and mythic demons, where the deceased ponder politics, religion, injustice, and war. With the encounters of Maali and the second-person narrative employed in the novel, Karunatilaka dissolves the boundary between the dead and the living, employing wit and dark satire to engage with historical violence, ethnic split, and the long, haunting shadow of Sri Lanka’s unquiet past.

### 3.2 Memory, trauma, and afterlife

In *The Seven Moons of Maali Almeida*, the protagonist, Maali, serves as a spectral guide, leading readers through his recollections of life and suffering in Sri Lanka during the civil war. The Sri Lankan Civil War (1983–2009) was a protracted and brutal conflict between the Sinhalese-dominated government and the Liberation Tigers of Tamil Eelam (LTTE), who sought an independent Tamil state. Rooted in postcolonial grievances and systemic discrimination, the conflict was marked by atrocities perpetrated by both sides, including mass disappearances, torture, and civilian massacres. The final phase of the war in 2009, particularly in the Vanni district, resulted in a catastrophic disruption of Tamil life. It affected both the political and cultural life of them. The postwar era transformed the Sri Lankan landscape into a haunting space, where memory was suppressed and victimhood politicised. As Rachel Seoighe says, “Tamil people, in order to preserve memory, took stones from the rubble of the graveyards after they were destroyed. The identity of persecution and victimhood persists and is manifest in these acts as a desire to commemorate the dead in the face of state erasure” (Seoighe, 2016). This struggle is to remember the deceased and to reclaim agency amidst state repression. It exemplifies how haunting functions both as a literary device and a political tool, which reveals hidden truths and challenges historical closure. The “In Between” space in this novel depicts the afterlife as a bureaucratic, chaotic realm that, in turn, shows Sri Lanka’s unresolved historical trauma. “All of these restless ghosts who have crowded the ‘In Between’ seem to be a metaphor for Sri Lanka” (Saha and Ghosh Sarbadhikary, 2024, p. 9). This haunting liminality is captured in Karunatilaka’s description: “All you see are spirits as anonymous and as forgotten as they were in life. And among the bombed, the burned and disappeared is you, cause of death as yet undetermined” (2022). The anonymity of these spirits mirrors the erasure of the disappearance in Sri Lanka’s political history. Maali’s interactions with the deceased, including victims of communal riots and civil war, assassinated journalists who exposed the truth, and disappeared activists who highlighted injustices, create a spectral narrative of Sri Lankan history, the untold. In this afterlife space, the dead traverse zones resembling Colombo and await judgement or reincarnation. Also, they are clinging to the injustices of their past lives, and remain unrestful because the truths of their lives and deaths remain obscured and unacknowledged, as well as manipulated by external forces. Karunatilaka juxtaposed the war trauma in this novel with a pinch of spectrality. As Thi et al. (2023) noted, “When these writers attempted to reconcile with their past, they plunged deeper into crises as it became clear that their memories were in fact nostalgia.”

Throughout the novel, Maali’s death is not a definitive end but a liminal, transitional phase, a space between worlds that defies finality. This temporal and existential suspension resonates with Shakespeare’s renowned line in *Hamlet* (1603), “The time is out of joint” (1.5.188), a phrase later referenced by Jacques Derrida in *Spectres of Marx* (1993) to depict the fragmented and nonlinear nature of both history and memory. For Maali, time is indeed “out of joint”: he inhabits the past, present, and future simultaneously. In the past, he was tormented by his experiences as a war photographer, witnessing scenes of mass killings, disappearances, and political assassinations that continue to haunt his consciousness. Today, he attempts to interfere in the affairs of the living from the dead, guiding his friends to the secret photos that contain the truth about his death and the abuses of influential political players. Tomorrow, he hangs on to the possibility that this truth, having been exposed, will spark political change and defy the culture of impunity. This stratified temporal presence corresponds to Gordon’s (1997) note that ghosts unsettle the “linearly temporal, and discrete spatiality of our conventional notions of cause and effect, past and present, conscious and unconscious” (p. 66). With Maali, such distinctions collapse through haunting, combining memory and action, testimony and prophecy. His spectral agency crosses temporal divides, proving that the past is never truly past; it forever continues to influence the present and the future, and through this spectral temporality, the novel challenges historical closure while underscoring the enduring nature of injustice and the moral obligation to bear witness beyond death. “Do you want to see your body? Do you want your life back? Or the real question, which you truly should be pondering. How the hell did you get here? You remember nothing, not pain, not surprise, not the last breath, nor where you took it” (Karunatilaka, 2022). Maali’s amnesia vocalises the collective condition of a nation haunted by what has been knowingly silenced.

The intergenerational and collective trauma serves as the animating force for Maali and the other spectres in *The Seven Moons of Maali Almeida.* It is trauma that is not just a personal affliction but rather a historical affliction, borne across communities and generations. And it is etched into landscapes, which are seared into the memories of survivors. Restorative justice is a valuable framework by critiquing the excessive psychologisation of trauma. It emphasises that wounds are not solely individual but are also collective and perpetuated within communities. “Restorative justice is not new to the country; it is very much a part of the Sri Lankan spiritual heritage of forgiveness, which understands that social development involves the transformation of individuals, not their prolonged imprisonment or death” (Dharmawardhane, 2013). The “In Between,” which is Karunatilaka’s conceptualisation of the spectral realm, reflects this spiritual heritage. Maali engages with memory as a moral imperative rather than pursuing karmic liberation. His haunting compels the living to confront acts of violence, recognise their complicity, and transform through the process of truth-telling, and this gives way to restorative justice. Karunatilaka shows how ghosts exemplifies this concept because they represent not isolated psyches but collective wounds that require communal acknowledgement. These spectral figures are refusing to leave so that their narratives can be conveyed to illustrate how trauma persists intergenerationally and influences both memory and identity in postwar Sri Lanka. A ghost imparts a profound truth to Maali, encapsulating the moral depravity of war as, “Evil is not what we should fear. Creatures with power acting in their own interest: that is what should make us shudder” (Karunatilaka, 2022, p. 25). Wars do not truly conclude; they persist until humanity’s thirst for blood and power is satiated. All too often, wars are initiated or perpetuated not for national security or principles but to divert attention from the abuses of those in power, and to cover up state atrocities behind a facade of patriotism. Who suffers? Not the political leaders who are orchestrating the bloodshed, but they remain unscathed in positions of safety and privilege. The victims are predominantly civilians: ordinary men, women, and children. They endure the destruction of their societies, the devastation of their economies, and death by bombings, disappearances, or starvation. From a restorative justice perspective, these ordinary victims constitute a silenced community. Their wounds require collective acknowledgement; without such recognition, the trauma festers and manifests as ghosts, protests, and suppressed memories. This destruction is associated with the realm of spectres in the novel to position the afterlife as a repository of political violence. And it acts as a domain where the dead continue to speak, recall, and testify. In doing so, Karunatilaka’s work resonates with Gordon’s (1997) assertion that haunting “always registers the harm inflicted or the loss sustained” (p. xvi). Thus, the afterlife in the novel is not an escape from history but its most intimate and inescapable confrontation. Maali’s own voice reflects this entanglement of death and memory: “I was there to witness. That is all. All those sunrises and all those massacres existed because I filmed them. Now, they are as dead as me” (Karunatilaka, 2022, p. 214). As a war photographer, Maali bore the burden of silent witnessing, documenting atrocities without the capacity to intervene. His camera captured truths that the living wished to cover up. In death, he realised that silence, whether imposed or self-imposed, amplifies violence. His experience testifies that the voices of the silenced civilians in the Sri Lankan civil war are unheard, and their mourning is rendered invisible.

### 3.3 The ghost being witness and archivist

*The Seven Moons of Maali Almeida* introduces the ghost of Maali as both a witness and an archivist of repressed histories, burdened with memory and trauma. As Gordon (1997) said, ghosts “appear when the trouble they represent and symptomise is no longer being contained or repressed” (p. 16). And posthumously, Maali discloses his cache of images. As a spirit, Maali witnesses not only his cruel murder but also decades of ethnic upheaval, which include state-backed disappearances and rebel atrocities. Restorative justice shows the acknowledgement of harm as something essential for a meaningful reconciliation. Offenders must first admit their wrongdoing before healing can occur. Nevertheless, in Karunatilaka’s Sri Lanka, neither state forces nor insurgent groups accept responsibility. Their refusal perpetuates cycles of silence. Thus, leaving ghosts as the sole agents demands acknowledgement. Here, haunting becomes a form of enforced recognition, as the unquiet dead represent harm denied in life. The victims of the 1983 riots, insurgencies, and enforced disappearances remain suspended in memory so that their suffering was never publicly acknowledged. As one ghostly voice insists, “We see you. We can make your killers pay. Justice will bring you peace. Your killers will beg for your mercy” (Karunatilaka, 2022). This shows the emphasis of restorative justice on acknowledging harm as a condition for healing. It is said because peace is impossible without recognition of wrongdoing. However, the novel also cautions against simple retribution, i.e., “Revenge is not a right. This island does not need more corpses. You are being a child” (Karunatilaka, 2022). Here, Karunatilaka aligns haunting more with restorative than with retributive justice. And underscores that justice to move beyond cycles of vengeance toward collective recognition and transformation. Karunatilaka thus illustrates how forgetting, framed as “moving on” or “forgiving,” is not neutral. But frames it as political, which is a strategy of erasure that continues violence. In contrast, the persistence of ghosts aligns with restorative justice principles. Whereas acknowledgement is not only ethical but also transformative, as it disrupts amnesia. It asserts that history cannot be healed without first being recounted. Maali’s struggle to preserve and reveal the truth of his photographs is a direct challenge to this enforced amnesia. His haunting of the living becomes an act of resistance against the institutional desire to erase or mystify uncomfortable histories. Thus, haunting is an unofficial legal process, a metaphorical court in which the dead serve as witnesses and judges of the living. The ghosts’ demand for recognition mirrors the testimonial capacity of truth commissions or restorative circles. Thus, it converts the spiritual into a sociological type of law. Karunatilaka thus reinterprets justice as conversation instead of punishment, by basing Buddhist metaphysics on the social mechanisms of accountability and healing.

The transitional state experienced by Maali resonates with Sri Lankan Buddhist cosmology. It is particularly connected with the concept of the antarabhava, the intermediate state between death and rebirth. While Orthodox Theravāda Buddhism, which is the predominant tradition in Sri Lanka, typically asserts that rebirth is immediate. The folk Buddhist practices and popular beliefs allow for a transitional period during which spirits linger and are burdened by unresolved karmic debts or attachments. Maali’s seven moons in the “In Between” reflect this cultural space, positioning him as a restless spirit akin to the preta (hungry ghost). And his unsatisfied craving (tanha) binds him to the realm of the living. The nation itself becomes a haunted space, where spectrality exposes the lingering effects of war and the failure of reconciliation. “Sri Lanka itself is purgatory for many, hanging between life and death or near death or after death” (John, 2022, *as cited in* Mathew, 2024). In Buddhist philosophy, such attachment is generally viewed as an impediment to liberation from samsara. However, in Maali’s case, his attachment to photographs and memories goes beyond the personal and becomes the political. By resisting the act of “forgetting,” Maali subverts the Buddhist ideal of non-attachment and transforms craving into a moral imperative to bear witness. This inversion illustrates how Buddhist discourse in post-war Sri Lanka has occasionally been appropriated. It promotes reconciliation through forgetting, and urging individuals to relinquish the past “for the sake of harmony,” thereby fostering political amnesia. This forgetting finds a spiritual corollary in Sri Lankan folk-Buddhist cosmology. Thus, ghosts (preta) who do not release unresolved attachment to either this or the other side become stuck in between, a condition commonly described as a result of karmic remainder and outstanding obligations. However, Maali’s attachments are not rooted in familial or material longing. But it is in his ethical refusal to abandon the truth about violence and injustice. This transforms his “clinging” (upādāna) into a radical act of moral persistence, which is typically seen in Buddhist teaching. His ghostly condition represents a threshold political stage where samsara is not accepted as inevitable but confronted by the return of what the living has tried to suppress.

As the novel itself makes clear, “Every soul is allowed seven moons to wander the In Between. To recall past lives. And then, to forget. They want you to forget. Because, when you forget, nothing changes” (Karunatilaka, 2022). Here, enforced amnesia appears not as spiritual release but as a political mechanism of silence. Karunatilaka reimagines the Buddhist afterlife as a battleground for historical truth. Consequently, Maali’s spectral presence transforms the intermediate state into a site of resistance. Thus, it challenges both karmic logic and the sociopolitical applications of Buddhism that advocate forgetting. His haunting therefore becomes not merely a metaphysical quest but also an exploration of how religion and power intersect within Sri Lanka’s memory space.

The lines “You want to ask the universe what everyone else wants to ask the universe. Why are we born, why do we die, why anything has to be. And all the universe has to say in reply is: I do not know, arsehole, stop asking” (Karunatilaka, 2022, p. 176), sums up the existential crisis faced by Maali as a ghost. Rather than providing closure, the afterlife presents a disordered world for Maali. In encapsulates the futility of imposing meaning on a system inherently designed to be indeterminate becomes apparent. Maali, as a witness, is burdened with both his own and others’ memories, which are vague and unresolved. This uncertainty mirrors the Buddhist concept of avidyā (ignorance), the initial link in the cycle of dependent origination (paticca-samuppāda). It perpetuates the cycle of birth, death, and rebirth (samsara). In Sri Lankan Theravāda Buddhism, existence and death are governed by the law of karma. Still, the reasons for one’s suffering or the timing of one’s death seldom appear clear. Instead, existence is perceived as inherently characterised by dukkha (unsatisfactoriness) and impermanence (anicca), leaving individuals without a definitive cosmic answer to the question “why.” Maali’s sarcastic portrayal of the universe’s indifference parodies this Buddhist acceptance of existential uncertainty, showing how, in both spiritual and political contexts, answers are often withheld, deferred, or obscured in mystery.

Maali perceives his role as a documentarian of violence with profound gravity. “Maali’s omnipresence after his death… does not come with omnipotence. His current state almost mirrors the position he had occupied as a war photographer… constrained to the role of a silent witness to the mayhem and massacres unleashed before him” (Saha and Ghosh Sarbadhikary, 2024). Maali’s ghostly voice resists erasure and reclaims agency. His spectral presence becomes a counter-narrative to dominant historical silences. If a photograph is worth a thousand words, then what significance do his photographs hold, which bear witness to crimes that words have either failed to document or have been compelled to silence? For Maali, these images transcend mere artistic expression. They represent fragments of suppressed history, visual evidence capable of challenging the meticulously constructed narratives of state, rebel, and even global powers. His haunting is thus not a passive wandering but a deliberate act which enlists, manipulates, and urges his living friends to complete the work he can no longer undertake himself. On top of these photographs, the voices of the ghosts themselves serve as a form of testimony. Their spectral narratives, akin to Maali’s images, operate as what restorative justice theorists term storytelling circles. And it portrays the venues where previously silenced experiences of harm are publicly articulated and acknowledged. Within the framework of restorative justice, storytelling transcends the mere recounting of trauma. It challenges hegemonic silences, facilitates the circulation of suppressed truths, and demands recognition. Consequently, Maali’s spectral archive is not only visual evidence but also a platform for the deceased to communicate with the living. This aligns with Gordon’s assertion that ghosts demand “something-to-be-done” (1997, p. xvi). And thus, each testimony obliges the listener to respond. Like restorative justice processes storytelling to acknowledge harm and initiate dialogue, Karunatilaka’s novel employs ghostly testimony. This testimony acts as a counter-history, a form of public acknowledgement that resists the state’s inclination for closure. As a ghost, he is liberated from the physical dangers associated with journalism. Yet he remains bound by a different urgency, the moral and ethical obligation to ensure that the truth does not perish with him. “By maintaining his distinctive voice… Maali’s ghost prevents the appropriation of his own tale. In addition, in doing so… ends up preserving a piece of history… never properly commemorated” (Saha and Ghosh Sarbadhikary, 2024). Thus, haunting is not passive, and it’s a form of resistance. He believes that publicizing these photographs can disrupt hegemonic discourses and create space for counter-memory. This haunting is psychological, political, and cosmological in nature. The novel portrays how ghosts, just like subordinated groups in society, show the way “a sense of inferiority provokes a sense of ownership that fosters a sense of resistance to the external commands or repetitive functions” (Kumar and Ajit, 2025). Politically, it stems from Sri Lanka’s colonial and postcolonial history, which is from the racialised hierarchies and communal fault lines that flourished under British colonialism. The photographs are engraved with these intertwined histories, serving as a spectral archive that cannot be erased.

From the perspective of Sri Lankan Buddhism, Maali’s actions complicate the traditional understanding of a ghost’s purpose. In Theravāda cosmology, beings in the preta realm often linger due to unresolved attachments or karmic debts. And because of this, liberation depends on relinquishing such ties. However, Maali’s “attachment” to photographs and their truth is not driven by greed, longing, or revenge; it is driven by an ethical responsibility to memory. This reconfigures the Buddhist concept of upādāna (clinging) into a form of resistance. Therefore, rather than clinging to individual loss, Maali clings to group justice.

The final part of the novel represents Maali’s realisation of moral and religious development from revenge to freedom. It is established in the novel where he moves toward and eventually passes the “River of Births.” Karunatilaka writes, “The river is not deep; you can feel the bottom with your toes… The water is as warm as the air is cool. You are not alone at this river; all around you are swimmers braving the currents and hugging the banks… You are not the you that you think you are… You are everything you have thought and done and been and seen” (2022). The river thus serves as both a metaphysical and ethical domain, a collective threshold where the individual and the community converge in acts of renewal. Previously, he acknowledged the futility of revenge as, “You no longer care if your photos are seen or not. Because Jaki and DD still have breath and, even though that will not make up for this whole damn mess, it is something. And, without a doubt, that is the kindest thing you can say about life. It’s not nothing.” (Karunatilaka, 2022). His haunting is resolved not through triumph but through surrender. It is an act of ethical renewal that resonates with restorative justice’s focus on acknowledgement instead of retribution. In this sense, Maali’s passage symbolises what transitional societies themselves must undertake. It gives the understanding that society needs to move from retributive rage to restorative dialogue, which is like moving from trauma to ethical coexistence. And Maali leaps toward transcendence as, “So, you jump. And when you jump you know three things. That the brightness of The Light will force you to open your eyes wider. That you will choose the same drink and it will take you somewhere new. And that, when you get there, you will have forgotten all of the above” (Karunatilaka, 2022). Maali embodies the potential for liberation through remembrance and release. The “River of Births” thus emerges as Karunatilaka’s ultimate metaphor for reconciliation. And hence the pursuit of justice is fulfilled not through punishment but through the courage to confront, forgive, and ultimately transcend the wounds that bind both the living and the deceased.

## 4 Conclusion

The “In Between” in *The Seven Moons of Maali Almeida* functions as a circle, which is spectral and chaotic in nature. It is a liminal space where the dead ‘speak,’ where silenced testimonies re-emerge, and where the living are compelled toward truth-telling and accountability. The haunting does not obliterate pain but insists on its recognition. And therefore, reminding both victims and perpetrators that forgetting equates not to freedom but to denial. Thus, the In Between may be interpreted as a metaphorical space of restorative justice, where the dead and the living—the victim and the abuser—engage in dialogue. Although reconciliation is not fully realised, the act of haunting itself becomes a demand for such a process. The ghosts resist closure; instead, they insist on radical remembrance as a prerequisite for justice. Also, the “River of Births” thereby becomes Karunatilaka’s final metaphor for reconciliation in which Buddhist karmic law simulates the restorative pursuit of moral balance. By locating spiritual transcendence and social repair together, the novel points toward a sociological imagination of justice that combines the ethical, the communal, and the legal.

In this way, the literature here becomes an essential site for testimony. Through the interweaving of memory, trauma, and the supernatural, the novel restores narrative agency to those historically silenced. Fiction acts as a counter-archive, a space where ghosts return not only to haunt others, but also to speak, disrupt, and demand justice. In life, Maali is fragmented by ethnicity, sexuality, and ideological politics. After death, Maali becomes a composite character whose liminality grants him insight, understanding, and subversive power. Maali, as a spectre, embodies uncompleted business, and he represents the voice of the ungrieved, the undocumented, and the denied. His haunting is politicised, and it’s not melancholic, but serves as a ghostly resistance to oblivion. *The Seven Moons of Maali Almeida* reveals how ghosts, rather than being remnants of superstition, are agents of history, memory, and justice. Karunatilaka emphasises the ethical imperative to heed what has been silenced by engaging dialogue between the dead and the living. In doing so, he underscores the enduring power of literature to challenge dominant narratives and create spaces for radical remembrance.

On the whole, the ghost in this narrative is not merely a vestige of the past but a political agent and archivist who challenges the institutional and cultural erasures that attempt to sanitise history. Maali’s endeavour to safeguard his collection of photographs opposes both political amnesia and the Buddhist directive to relinquish attachment, thereby transforming clinging into a moral imperative of testimony. In doing so, Karunatilaka underscores the ambivalence of Buddhism in Sri Lanka, simultaneously a philosophy of liberation and, in its politicised forms, a discourse that has occasionally been employed to legitimise silence, forgetting, or Sinhala-Buddhist majoritarian dominance in Sri Lankan politics. By reinterpreting the Buddhist concept of the intermediate state (antarabhava) as a liminal political space, the novel posits that forgetting is not liberation but complicity, and that remembering is not bondage but resistance. Through Maali’s spectral presence, Karunatilaka positions haunting as both a metaphor and a method. It’s a metaphor for the enduring trauma of civil war, state terror, and ethnic violence, and a method for narrating the silenced and repressed histories that resist closure. The afterlife in *The Seven Moons of Maali Almeida* thus becomes an alternative archive where ghosts contain unresolved suffering and demand accountability. All things considered, Maali’s haunting affirms that the past cannot be buried and it persistently resurfaces to unsettle the present and shape the future. By blending satire, the trauma narrative, and Buddhist cosmology, Karunatilaka transforms ghosts into figures of ethical resistance and thus reminds us that justice begins with the refusal to forget. As Karunatilaka poignantly writes, “They say the truth will set you free, though in Sri Lanka the truth can land you in a cage. And you have no more use for truth or cages or killers or lovers with perfect skin. All you have left are your images of ghosts. That may well be enough” (Karunatilaka, 2022). The truth may endanger living, but in the spectral archive of haunting and photographs, it endures as a form of resistance.

## Data Availability

The original contributions presented in the study are included in the article/supplementary material, further inquiries can be directed to the corresponding author.
